# Primary Human Trophoblasts Mimic the Preeclampsia Phenotype after Acute Hypoxia–Reoxygenation Insult

**DOI:** 10.3390/cells11121898

**Published:** 2022-06-11

**Authors:** Barbara Fuenzalida, Sampada Kallol, Jonas Zaugg, Martin Mueller, Hiten D. Mistry, Jaime Gutierrez, Andrea Leiva, Christiane Albrecht

**Affiliations:** 1 Institute of Biochemistry and Molecular Medicine, Faculty of Medicine, University of Bern, 3012 Bern, Switzerland; barbara.fuenzalida@ibmm.unibe.ch (B.F.); sampuak@gmail.com (S.K.); jonas.zaugg@ibmm.unibe.ch (J.Z.); 2 Swiss National Center of Competence in Research, NCCR TransCure, University of Bern, 3012 Bern, Switzerland; 3 Department of Obstetrics and Gynaecology, Inselspital, University of Bern, 3012 Bern, Switzerland; martin.mueller@insel.ch; 4 Department of Women and Children’s Health, School of Life Course and Population Health Sciences, King’s College London, London WC2R 2LS, UK; hiten.mistry@kcl.ac.uk; 5 School of Medical Technology, Faculty of Medicine and Sciences Health, Universidad San Sebastián, Santiago 7510157, Chile; jaime.gutierrez@uss.cl (J.G.); andrea.leiva@uss.cl (A.L.)

**Keywords:** preeclampsia, hypoxia, hypoxia/reoxygenation, sFlT-1/PlGF ratio, primary trophoblast, inflammation, oxidative stress

## Abstract

Preeclampsia (PE) is a pregnancy-specific disorder that affects 3 to 5% of pregnancies worldwide and is one of the leading causes of maternal and fetal morbidity and mortality. Nevertheless, how these events occur remains unclear. We hypothesized that the induction of hypoxic conditions in vitro in primary human trophoblast cells would mimic several characteristics of PE found in vivo. We applied and characterized a model of primary cytotrophoblasts isolated from healthy pregnancies that were placed under different oxygen concentrations: ambient O_2_ (5% pCO_2_, 21%pO_2_, 24 h, termed “normoxia”), low O_2_ concentration (5% pCO_2_, 1.5% pO_2_, 24 h, termed “hypoxia”), or “hypoxia/reoxygenation” (H/R: 6 h intervals of normoxia and hypoxia for 24 h). Various established preeclamptic markers were assessed in this cell model and compared to placental tissues obtained from PE pregnancies. Seventeen PE markers were analyzed by qPCR, and the protein secretion of soluble fms-like tyrosine kinase 1 (sFlT-1) and the placenta growth factor (PlGF) was determined by ELISA. Thirteen of seventeen genes associated with angiogenesis, the renin–angiotensin system, oxidative stress, endoplasmic reticulum stress, and the inflammasome complex were susceptible to H/R and hypoxia, mimicking the expression pattern of PE tissue. In cell culture supernatants, the secretion of sFlT-1 was increased in hypoxia, while PlGF release was significantly reduced in H/R and hypoxia. In the supernatants of our cell models, the sFlT-1/PlGF ratio in hypoxia and H/R was higher than 38, which is a strong indicator for PE in clinical practice. These results suggest that our cellular models reflect important pathological processes occurring in PE and are therefore suitable as PE in vitro models.

## 1. Introduction

Preeclampsia (PE) is the most common pregnancy complication and one of the three leading causes of maternal morbidity and mortality worldwide, complicating around 2–8% of pregnancies [1]. PE is characterized by hypertension (systolic blood pressure ≥ 140 mm Hg and diastolic blood pressure ≥ 90 mm Hg) and proteinuria (≥300 mg/24 h) from 20 weeks of gestation [2]. A critical factor in PE is a poor placental invasion of the uterine vasculature by trophoblast cells and poor differentiation, which leads to impaired placental perfusion, oxidative stress, cellular damage, inflammation, vascular dysfunction, and the release of the antiangiogenic factors soluble fms-like tyrosine kinase 1 (sFlT-1) and soluble endoglin (sENG) components into the maternal circulation [3,4,5,6,7,8]. It has been observed that the increase in oxidative stress from high ROS production and lipid peroxidation, disturbances in the renin–angiotensin system (RAS), immune maladaptation, and genetic susceptibility may all contribute to the pathogenesis of PE [5,9,10,11,12,13]. Nuclear factor erythroid 2-related factor (NRF2) is the master regulator of cellular oxidative stress responses. It has been associated not only with the pathology of PE but also with the pathogenesis of other disorders such as cancer, Alzheimer’s disease, and articular disease [14,15,16,17].

In addition, an increase in different inflammation markers in PE has been described, including interleukin (IL)-1β and caspase 1 (CASP1), as well as the activation of the Nod-like receptor protein 3 (NLRP3) inflammasome [18,19,20].

Based on the findings in different animal and cellular models as well as in preeclamptic patients, some of the differentially expressed proteins were proposed as characteristic markers of PE. These proteins are associated with angiogenic imbalance (FlT-1, PlGF, ENG, and VEGF A), RAS receptors (AGTR1, AGTR4), stress markers (HIF1α, NRF2), endoplasmic reticulum stress (GRP78, GRP94), placenta-specific proteins (PP13, PAPP-A, and RGC-32), and the inflammasome complex (NLRP3, IL-1β, and CASP1). 

In the first trimester of pregnancy, low oxygen partial pressures (pO_2_) are vital for placental development [21]. This stimulus is required for cytotrophoblast proliferation and differentiation along the extravillous trophoblast (EVT) pathway, as well as for regulation of the invasion of EVT trophoblasts, which is required for uterine arteries’ remodeling. During pregnancy, low oxygen partial pressures (<2% pO_2_) are observed in the first trimester, which increase to <8% pO_2_ as pregnancy progresses during middle and late gestation [22,23]. Dysregulation in this process, such as persistent low O_2_ concentrations (hypoxia) or cycles of hypoxia–reoxygenation can lead to pregnancy complications such as PE and intrauterine growth restriction (IUGR) [22,23,24]. PE, however, is a complex and multifactorial disease associated with an abnormal differentiation of the trophoblast to the invasive EVT occurring in the first trimester. This results in a shallow placental invasion and a failure of the uteroplacental perfusion, triggering the release of antiangiogenic factors to the maternal circulation, damaging the maternal vascular endothelium, and generating a widespread maternal endothelial dysfunction. It is well accepted that several mechanisms contribute to the pathogenesis of PE. However, it is unclear whether the pathways involved in PE are interrelated, have synergistic effects, or act independently. 

Since abnormal O_2_ concentrations were associated with the dysregulated development and function of the human placenta in PE, we hypothesized that the induction of hypoxic conditions in primary human trophoblasts would mimic several characteristics of PE. Hence, we established a PE model in primary human trophoblast cells obtained from full-term healthy placentae by inducing hypoxia and/or hypoxia/reoxygenation (H/R) and evaluated the cell biological factors dysregulated in PE. Moreover, we assessed the gene expression of various well-established PE markers in these trophoblast cell models, i.e., under ambient O_2_ concentration (termed “normoxia”), persistent low O_2_ concentration (termed “hypoxia”), and H/R, and compared the expression patterns with placental tissues collected from PE pregnancies.

## 2. Materials and Methods

### 2.1. Study Group

Human placentae were obtained from full-term normal pregnancies (38–40 weeks) from the Division of Gynecology and Obstetrics of the Lindenhofgruppe, Bern, Switzerland (*n* = 9) and from women with PE (32–40 weeks) and full-term normotensive controls (38–40 weeks) pregnancies from Hospital Clínico UC CHRISTUS (*n* = 29). PE was defined as a systolic blood pressure > 140 mm Hg and a diastolic blood pressure > 90 mm Hg, determined on 2 occasions >4 h apart and arising after 20 weeks of gestation in a previously normotensive woman and de novo proteinuria (protein: creatinine ratio (PCR) > 30; urine protein concentration > 3 g/L in 2 random clean-catch midstream specimens collected >4 h apart) with no evidence of urinary tract infection [2]. The collection of samples was performed according to the principles outlined in the Declaration of Helsinki. All procedures were approved by the local ethics committee (Canton of Bern, Switzerland Basec Nr.2016-00250) and the Faculty of Medicine at Pontificia Universidad Católica de Chile (PUC, ID-180810004). Informed consent and clinical data from patients were obtained. General maternal (i.e., age, height, weight, and blood pressure) and neonatal (i.e., sex, gestational age, weight, height, and the weight of the placenta) variables were obtained from the clinical records.

### 2.2. Primary Human Trophoblast Cells

Primary cytotrophoblast cells (CTB) were isolated from healthy term placenta villous tissues. In brief, 30 g of placental tissue was carefully minced and digested three times for 30 min at 37 °C in saline Hanks/HEPES solution plus DNase I (Sigma Aldrich, St. Louis, MI, USA) and trypsin (Thermo Fisher Scientific, Waltham, MA, USA), as described previously [25]. After isolation, the cellular pellets were resuspended in DMEM and separated by centrifugation (1500 RCF, 20 °C, 20 min) using a 10–70% Percoll gradient (Sigma Aldrich, St. Louis, MI, USA). CTB cells were obtained from gradient fractions between 35 and 55% Percoll. Cells were plated at a density of 200,000 cell/cm^2^ in DMEM high glucose (4.5 g/L glucose) medium supplemented with 10% fetal bovine serum and 1× antibiotic-antimycotic (Gibco, Waltham, MA, USA). After isolation, the cells were cultured for 12 h to allow them to attach before being exposed to different oxygen conditions.

The purity of isolated CTB was evaluated by staining with specific cells markers (an antibody cocktail containing either anti-cytokeratin 7 (mean 93.4% ± 2.01 positivity) direct labeled with Alexa Fluor 488^®^ (Novus Biologicals, Englewood, CO, USA) plus anti-vimentin (mean 2.44% ± 0.87 positivity) labeled with Alexa Fluor 647^®^ (Novus Biologicals, Englewood, Co, USA) or anti-von Willebrand factor (mean 0.28% ± 0.06 positivity) marked with Alexa Fluor 647^®^ (Novus Biologicals, Englewood, Co, USA)). Cells were acquired by flow cytometry (BD FACS LSRII; BD Biosciences, Franklin Lakes, NY, USA). Data acquisition for each staining was based on at least 10,000 events and performed using BD FACSDiva™ software (BD Biosciences, Franklin Lakes, NJ, USA), and the data were analyzed using FlowJo^®^ software version 10 (FlowJo LLC, Ashland, OR, USA).

### 2.3. Models of Preeclampsia

CTB isolated from healthy term placentae were cultured as described above for 12 h and subsequently kept either at ambient O_2_ concentration, defined as a control condition and termed “normoxia” (5% pCO_2_, 21% pO_2_, 74% N_2_, 24 h), at low O_2_ concentration, termed as “hypoxia” (5% pCO_2_, 1.5% pO_2_, 93.5% N_2_, 24 h), or at H/R (6 h intervals alternating between normoxia and hypoxia for 24 h) [26]. 

### 2.4. Isolation of mRNA and Quantitative RT-PCR 

Total RNA was extracted from placental tissues (PE and normotensive control, 100 mg of tissue was homogenized) and CTB after 24 h using TRI reagent (Invitrogen, Oxford, UK). Then, 1 μg of total RNA was reverse transcribed to cDNA using the GoScript™ Reverse Transcriptase System (Promega, Madison, Wi, USA), according to the manufacturer´s instructions. Quantitative reverse transcription-PCR (qRT-PCR) was performed, as previously described [27]. In brief, qRT-PCR was carried out on a CFX qRT-PCR System using the SYBR^®^ Green PCR master mix detection kit (Promega, Madison, Wi, USA). The primer pairs are listed in Table 1. The relative gene expression was calculated using the 2^−^^ΔΔCq^ method, using Tyrosine 3-monooxygenase/tryptophan 5-monooxygenase (*YWHAZ*) as the reference gene.

### 2.5. Lipid Peroxidation

As a marker of lipid peroxidation, malondialdehyde (MDA) formation was measured using thiobarbituric acid reactive substances (TBARS) assays in cell culture media, as described in [28]. In brief, cell culture media were collected between 6 and 24 h during normoxia, hypoxia, and H/R conditions. The MDA standard curve was prepared for 0.007–2 nmol/mL. Then, 15% *w*/*v* trichloroacetic acid (TCA, Merck, Darmstadt, Germany), sample or MDA standard (1,1,3,3 tetraethoxypropane), and 0.67% *w*/*v* thiobarbituric acid (TBA, VWR, Dietikon, Switzerland) in 2.5 M HCl were mixed in a 4:5:8 ratio, vortexed, and boiled for 20 min at 95 °C. After cooling the samples to room temperature, 1-butanol was added and gently mixed. (Sigma Aldrich, St. Louis, MI, USA). For phase separation, the samples were centrifuged at 1000 RCF for 1 min. Then, 200 μL of the organic phase was transferred to a black-wall 96-well plate and measured using a Flex Station II fluorescence microplate reader (Thermo Fisher Scientific, Waltham, MA, USA). The MDA combined with TBA to form a fluorescent adduct detected at an excitation/emission wavelength of 530/550 nm. The MDA equivalents were calculated by interpolation to the standard curve.

### 2.6. Measurement of Protein Secretion by ELISA

The secretion of β-hCG, sFlT-1, and PlGF into CTB culture media after 6–24 h exposure to normoxia, hypoxia, and H/R was measured by using a human hCG (intact) ELISA kit (RAB0092, Sigma Aldrich, St. Louis, MI, USA) and sFlT-1 and PlGF ELISA kits (EA100379 and EA100342, respectively, OriGene, Rockville, MD, USA) following the manufacturer’s instructions. The consecutive absorbance measurement was carried out at 450 nm on a Vmax microplate reader (Molecular device, San Jose, CA, USA). The concentrations of hCG, sFlT-1, or PlGF released by CTB were interpolated using the respective standard curves.

### 2.7. Statistical Analysis

The values for the maternal and neonatal characteristics are presented as the mean ± SD, as described previously [29]. The relative gene expression and protein secretion were presented as the mean ± S.E.M. for the experiments, where *n* indicates the number of different individual isolated from the CTB cultures or individual placentae. Two or more groups were compared using the student’s *t*-test or ANOVA, respectively. *p* < 0.05 was considered statistically significant. Graphpad Prism 9.2 (GraphPad Software Inc., San Diego, CA, USA) was used for data analysis and for creating the figures.

## 3. Results

### 3.1. Maternal and Neonatal Variables in PE and Normotensive Control Pregnancies

The women with normotensive control pregnancy or PE were of comparable age, height, preconceptional weight, delivery, and basal glycemia (Table 2). The systolic and diastolic arterial blood pressure in PE patients was higher than in the normotensive controls. Women with PE presented proteinuria (>300 mg/24 h). Neonates from women with PE had a lower gestational age, birth weight, height, and ponderal index compared with the neonates from normal pregnancies (Table 2). Although the birthweight of the babies born to women with PE were lower, they did not constitute IUGR.

### 3.2. In Vitro Models Reflect the Angiogenic and RAS Receptor Expression Patterns Found in PE

**Figure 1:**
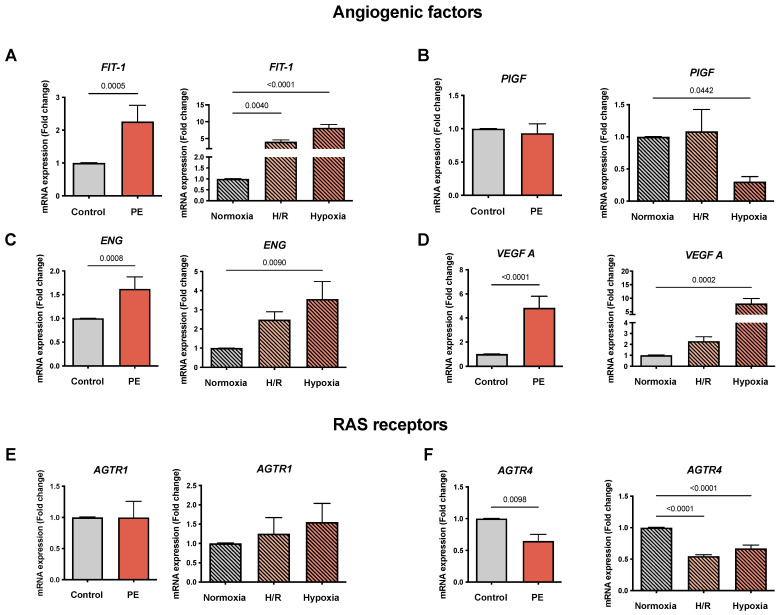
*The mRNA expression of genes involved in angiogenesis and the renin–angiotensin system (RAS) reflects a preeclampsia (PE) profile*. The mRNA abundance of (**A**) FlT-1, (**B**) PlGF, (**C**) ENG, (**D**) VEGF A, (**E**) AGTR1, and (**F**) AGTR4 in placental tissues from normotensive controls (gray bar; *n* = 16) and preeclamptic (PE: orange bar; *n* = 13) pregnancies is shown. The cultured primary cytotrophoblasts (CTB) were exposed either to normoxia (24 h: gray bar with diagonal lines; *n* = 9), hypoxia/reoxygenation (H/R; 6 h intervals each of normoxia and hypoxia for 24 h; light orange bar with diagonal lines; *n* = 9) and hypoxia (24 h; dark orange bar with diagonal lines; *n* = 9). The procedures regarding the processing of the placental tissues as well as the isolation of the CTB from the healthy term placentae are described in Materials and Methods. The gene expression was assessed by real-time PCR and normalized to the reference gene YWHAZ. Data are shown as the mean ± S.E.M. and represent the fold change (2^−^^ΔΔCq^) of the mRNA expression in comparison to the control placentae and normoxia, respectively. The placental expression patterns between the control and PE were compared using the student’s t-test. For comparison of normoxia with hypoxia and H/R, ANOVA was applied. *p* < 0.05 was considered statistically significant. FlT-1: fms-like tyrosine kinase 1; PlGF: placental growth factor; ENG: endoglin; VEGF: vascular endothelial growth factor; ATR: angiotensin receptor; YWHAZ: tyrosine 3-monooxygenase/tryptophan 5- monooxygenase activation protein zeta.

We analyzed the mRNA expression of *FlT1*, *ENG*, *VEGF A* (reported to be elevated in PE), and *PlGF* (decreased in PE) in preeclamptic and healthy placentae and in the PE cell models (normoxia, H/R, and hypoxia). As expected, *FlT-1* was increased in PE placentae compared to the controls (2.26 ± 0.49 vs. 1.01 ± 0.01, fold change (FC); *p* = 0.0005), as well as in H/R and hypoxia compared to normoxia (4.07 ± 0.52 and 8.18 ± 1.02 vs. 1.01 ± 0.01 FC, respectively; *p*= 0.004 and *p* < 0.0001) (Figure 1A). The placentae from women with PE showed no differences in *PlGF* expression, and no significant differences were found in H/R (Figure 1B). However, in hypoxia a decrease in *PlGF* compared to normoxia (0.30 ± 0.08 vs. 1.01 ± 0.01 FC; *p* = 0.0442) was observed (Figure 1B). *ENG* was increased in the PE placentae (1.62 ± 0.25 vs. 1.01 ± 0.01 FC; *p* = 0.0008) and in both hypoxic cell models compared to normoxia (3.56 ± 0.91 vs. 1.01 ± 0.01 FC; *p* = 0.009), but only hypoxia alone reached statistical significance (Figure 1C). Similarly, for *VEGF A* we observed an increase in PE placentae (4.83 ± 0.87 vs. 1.01 ± 0.01 FC; *p* < 0.0001) and the hypoxic cell models compared to normoxia, but only hypoxia treatment alone resulted in statistically significant changes (8.04 ± 1.82 vs. 1.01 ± 0.01 FC; *p* = 0.0002) (Figure 1D). 

Our study assessed the mRNA expression of the angiotensin receptors, *AGTR1* and *AGTR4,* in the established PE models. The RAS receptor *AGTR1* placental expression was neither altered in our PE cohort nor in the cell models of hypoxia and H/R (Figure 1E). *AGTR4* was reduced in the PE placentae compared to the controls (0.65 ± 0.1 vs. 1.01 ± 0.01 FC; *p* = 0.0098) as well as in H/R and hypoxia compared to normoxia (0.55 ± 0.03 and 0.67 ± 0.05 vs. 1.01 ± 0.01 FC, respectively; *p* < 0.0001 for both) (Figure 1F).

### 3.3. Hypoxia, Oxidative Stress, and Endoplasmic Reticulum Stress Markers Are Selectively Altered in the PE Cell Models 

**Figure 2:**
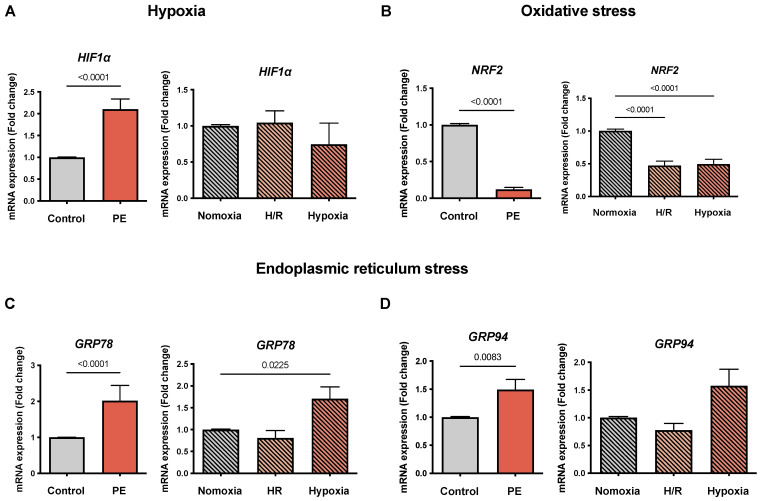
*The mRNA expression ofHIF1α, NRF2, GRP78, and GRP94 is selectively altered in the preeclampsia (PE) cell models.* The mRNA abundance of (**A**) hypoxia-inducible factor 1 alpha (HIF1α), (**B**) oxidative stress marker nuclear factor-erythroid 2-related factor 2 (Nrf2), (**C**) endoplasmic reticulum stress marker 78 kDa glucose-regulated protein (GRP78), and (**D**) 94 kDa glucose-regulated protein (GRP94) is shown in the placental tissues of the controls (gray bar) and preeclamptic (PE, orange bar) pregnancies. The cultured CTB were placed under normoxia (24 h, gray bar with diagonal lines), H/R (6 h intervals each of normoxia and hypoxia for 24 h; light orange bar with diagonal lines), and hypoxia (24 h; dark orange bar with diagonal lines). The procedures regarding the processing of the placental tissues as well as the isolation of the CTB from the healthy term placentae are described in Materials and Methods. The gene expression was assessed by real-time PCR and normalized to the reference gene *YWHAZ*. Data are shown as the mean ± S.E.M. (*n* = 16 Controls; 13 PE; 9 CTB cultured (normoxia, H/R, and hypoxia)) and represent the fold change (2^−^^ΔΔCq^) of the mRNA expression as compared to the control or normoxia. The placental expression patterns between the control and PE were compared using the student’s *t*-test. For comparison of normoxia with hypoxia and H/R, ANOVA was applied. *p* < 0.05 was considered statistically significant.

To determine the markers of hypoxia and oxidative stress in the cell model, we evaluated the *HIF1α* and *NRF2* mRNA levels. Similar to that reported in the literature, *HIF1α* was increased in the PE placentae compared to the controls (2.11 ± 0.23 vs. 1.01 ± 0.01 FC; *p* < 0.0001). In contrast, no significant differences were observed in H/R and hypoxia compared to normoxia (Figure 2A). *NRF2* was reduced in the PE placentae (0.12 ± 0.03 vs. 1.01 ± 0.01 FC; *p* < 0.0001) as well as in the cell models of H/R and hypoxia compared to normoxia (0.47 ± 0.07 and 0.5 ± 0.07 vs. 1.01 ± 0.01 FC, respectively; *p* < 0.0001 for both) (Figure 2B). 

We also evaluated selected markers of ER stress, namely *GRP78* and GRP94, which were previously reported to be increased in PE. In our study, *GRP78* placental expression was increased in PE compared to normotensive placentae (2.02 ± 0.42 vs. 1.01 ± 0.01 FC; *p* < 0.0001) and in hypoxia compared to normoxia in cells (1.71 ± 0.27 vs. 1.01 ± 0.01 FC; *p* = 0.0225) (Figure 2C). The same trend was found for *GRP94* (Figure 2D).

### 3.4. mRNA Expression of Placenta-Specific Proteins and Cell Cycle Regulators in PE Placentae and PE Cell Models

**Figure 3:**
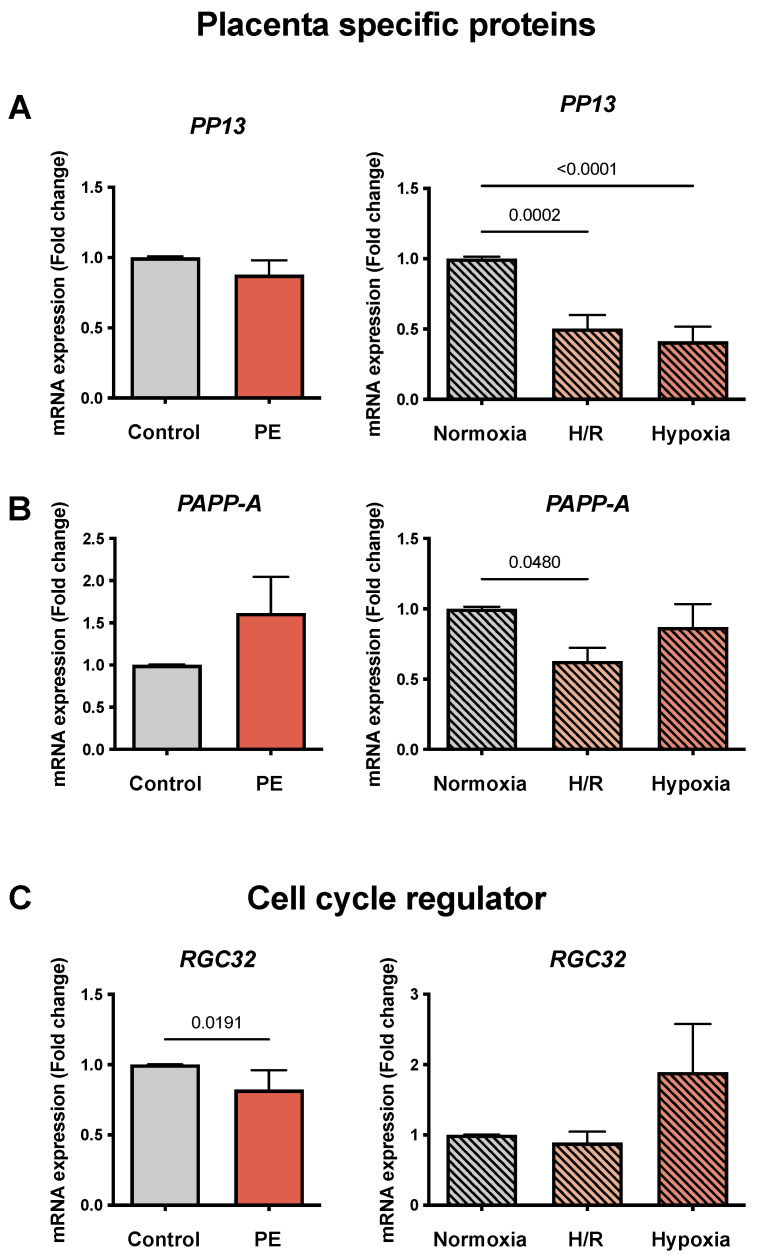
*The mRNA abundance of placenta-specific proteinsPP13, PAPP-A, and cell cycle regulation RGC32 is altered in the preeclampsia (PE) cell models*. (**A**) Placental protein 13 (PP13), (**B**) pregnancy-associated plasma protein-A (PAPP-A), and (**C**) the response gene to complement 32 (RGC32), a gene involved in cell cycle regulation. The placental tissues of normotensive controls (gray bar; *n* = 16) and women with PE (orange bar; *n* = 13) were compared. The cultured CTB were placed under normoxia (24 h; gray bar with diagonal lines; *n* = 9), H/R (6 h intervals each of normoxia and hypoxia for 24 h; light orange bar with diagonal lines; *n* = 9), and hypoxia (24 h; dark orange bar with diagonal lines; *n* = 9). The procedures regarding the processing of the placental tissues as well as the isolation of the CTB from the healthy term placentae are described in Materials and Methods. The gene expression was assessed by real-time PCR and normalized to the reference gene YWHAZ. Data are shown as the mean ± S.E.M. and represent the fold change (2^−^^ΔΔCq^) of the mRNA expression compared to the control or normoxia. The placental expression patterns between the control and PE were compared using the student’s t-test. For comparison of normoxia with hypoxia and H/R, ANOVA was applied. *p* < 0.05 was considered statistically significant.

To determine markers of placenta-specific proteins, we evaluated the mRNA expression of *PP13* and *PAPP-A*, which were described to be decreased in PE. The placentae from women with PE showed no differences in PP13 expression compared to the normotensive controls. In contrast, in the cell models, PP13 mRNA abundance was reduced in H/R and hypoxia compared to normoxia (0.5 ± 0.03 and 0.41 ± 0.11 vs. 1.01 ± 0.01 FC, respectively; *p* = 0.0002 and *p* < 0.0001) (Figure 3A). No significant differences for PAPP-A were found between the PE and normotensive placentae (Figure 3B). *PAPP-A* in H/R was diminished compared to normoxia (0.63 ± 0.1 vs. 1.01 ± 0.01 FC; *p* = 0.048), but no significant changes were found in hypoxia (Figure 3B). 

Following the trend reported in the literature, the cell cycle regulator *RGC32* was decreased in the PE placentae compared to the controls (0.82 ± 0.02 vs. 1.01 ± 0.01 FC; *p* = 0.0191), but no significant differences were observed in H/R and hypoxia in comparison to normoxia (Figure 3C).

### 3.5. Inflammasome Complex Is Altered in PE Placentae and in PE Cell Models

**Figure 4:**
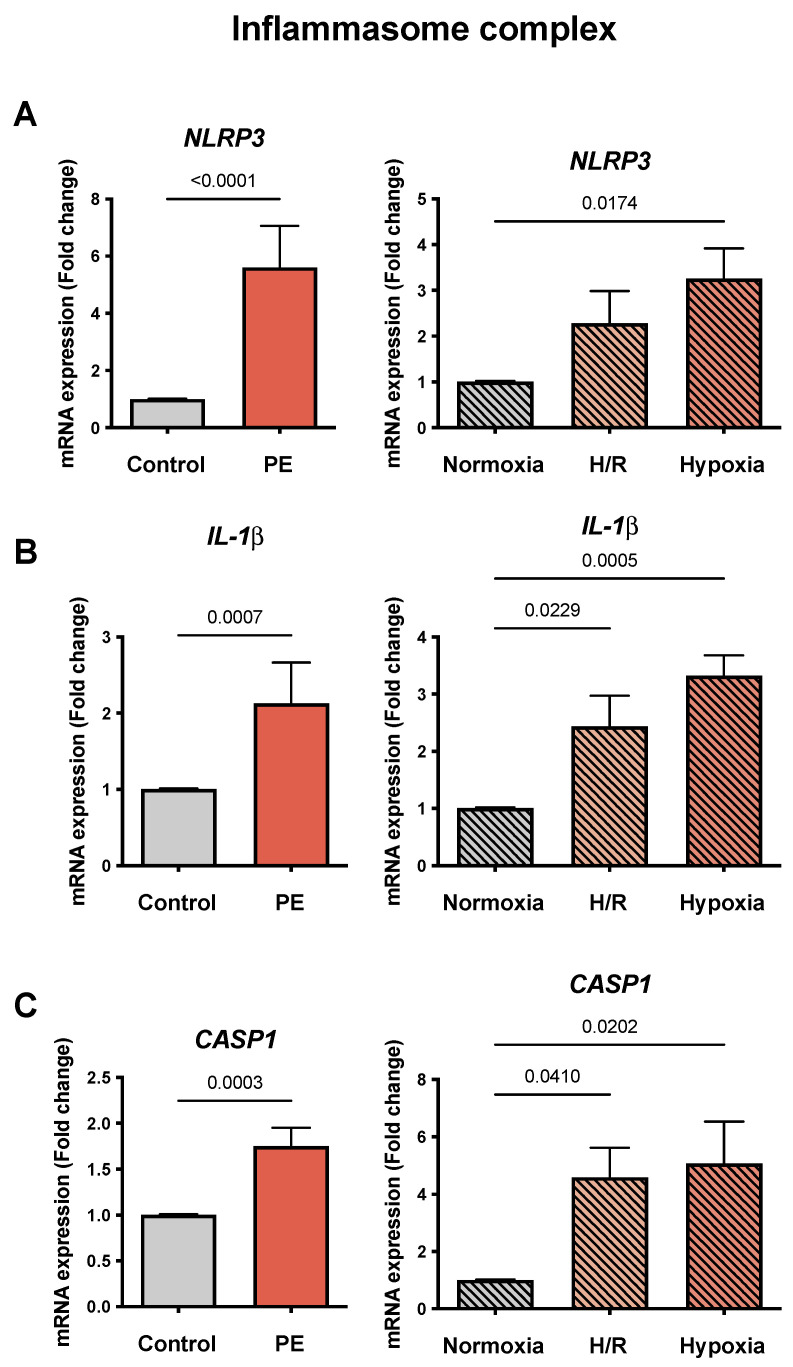
*The mRNA abundances of genes related to the inflammasome complex in the preeclampsia (PE) models reflect the expression patterns of PE placentae*. The gene expressions of (**A**) Nod-like receptor protein 3 (NLRP3), (**B**) interleukin 1β (IL-1β), and (**C**) caspase **1** (CASP1) were assessed by real-time RT-PCR and normalized to the reference gene YWHAZ. The placental tissues of the controls (gray bar; *n* = 16) and preeclamptic (PE, orange bar; *n* = 13) pregnancies were compared. The cultured CTB were placed under normoxia (24 h: gray bar with diagonal lines; *n* = 9), H/R (6 h intervals each of normoxia and hypoxia for 24 h; light orange bar with diagonal lines; *n* = 9), and hypoxia (24 h; dark orange bar with diagonal lines; *n* = 9). The procedures regarding the processing of placental tissues as well as the isolation of the CTB from the normotensive control placentae are described in Materials and Methods. Data are shown as the mean ± S.E.M. and represent the fold change (2^−^^ΔΔCq^) of the mRNA expression compared to the control or normoxia, respectively. The placental expression patterns between the control and PE were compared using the student’s *t*-test. For comparison of normoxia with hypoxia and H/R, ANOVA was applied. *p* < 0.05 was considered statistically significant.

To evaluate the markers of inflammation in PE, we measured the mRNA expression of three molecules of the inflammasome complex. *NLRP3* was increased in PE compared to the control placentae (5.61 ± 1.44 vs. 1.01 ± 0.01 FC; *p* < 0.0001) and in both hypoxic cell models compared to normoxia (3.26 ± 0.66 vs. 1.01 ± 0.01 FC; *p* = 0.0174), but only hypoxic treatment alone reached statistical significance (Figure 4A). *IL-1β* was increased in the PE placentae compared to the controls (2.13 ± 0.53 vs. 1.01 ± 001 FC; *p* = 0.0007), as well as in H/R and hypoxia compared to normoxia (2.44 ± 0.53 and 3.32 ± 0.36 vs. 1.01 ± 0.01 FC, respectively; *p* = 0.0229 and *p* = 0.0005) (Figure 4B). Similarly, *CASP1* was elevated in the PE compared to the control placentae (1.75 ± 0.20 vs. 1.01 ± 0.01 FC; *p* = 0.0003) and in both hypoxic cell models compared to normoxia (4.59 ± 1.02 and 5.07 ± 1.46 vs. 1.01 ± 0.01 FC, respectively; *p* = 0.0410 and *p* = 0.0202) (Figure 4C).

### 3.6. Human Chorionic Gonadotrophin Is Altered in PE Placentae and the PE Cell Models

**Figure 5:**
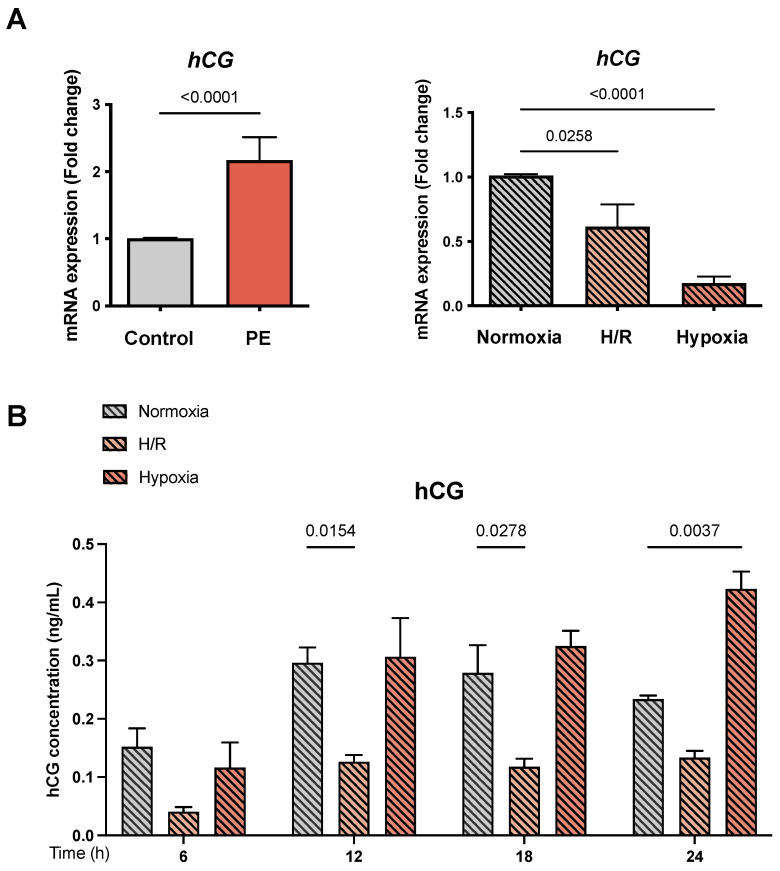
*The mRNA abundance and secretion of hCG.* (**A**) The expression of human chorionic gonadotrophin (hCG) as an indicator for syncytium formation was determined on an mRNA level by qRT-PCR. (**B**) The secretion of the hCG protein was analyzed during a time course of 6–24 h using ELISA. The placental tissues of the normotensive controls (gray bar; *n* = 16) and women with PE (orange bar; *n* = 13) pregnancies were compared. The primary cytotrophoblasts (CTB) were placed under normoxia (24 h: gray bar with diagonal lines; *n* = 9), H/R (6 h intervals each of normoxia and hypoxia for 24 h; light orange bar with diagonal lines; *n* = 9), and hypoxia (24 h; dark orange bar with diagonal lines; *n* = 9). The gene expression was assessed by real-time PCR and normalized to the reference gene YWHAZ. ELISA was performed as described in Material and Methods. Data are shown as the mean ± S.E.M. and represent the fold change (2^−^^ΔΔCq^) of the mRNA expression as compared to the control or normoxia. The placental expression patterns between the control and PE were compared using the student’s *t*-test. For comparison of normoxia with hypoxia and H/R, ANOVA was applied. *p* < 0.05 was considered statistically significant.

We quantified the effect of hypoxia and H/R on the mRNA expression and protein secretion of *hCG* as an indicator for cell differentiation and syncytium formation. We observed an increased *hCG* mRNA expression in the PE placental tissue compared to the controls (2.17 ± 0.34 vs. 1.01 ± 0.01 FC; *p* < 0.0001) (Figure 5A). In contrast, a decrease in *hCG* expression under H/R and hypoxia compared to normoxia was detected (0.61 ± 0.17 and 0.18 ± 0.01 vs. 1.01 ± 0.01 FC, respectively; *p* = 0.0258 and *p* < 0.0001) (Figure 5A). Furthermore, we evaluated the secretion of hCG protein during a 6–24 h time course under the different oxygen conditions. Reduced hCG secretion levels between H/R and normoxia were found after 12 h and 18 h (0.13 ± 0.03 and 0.12 ± 0.04 vs. 0.30 ± 0.07 and 0.28 ± 0.13, respectively; *p* = 0.0154 and *p* = 0.0278) (Figure 5B). hCG secretion was increased after 24 h in hypoxia compared to normoxia (0.42 ± 0.08 vs. 0.23 ± 0.01; *p* = 0.0037)) (Figure 5B).

### 3.7. Lipid Peroxidation in PE Cell Models

**Figure 6:**
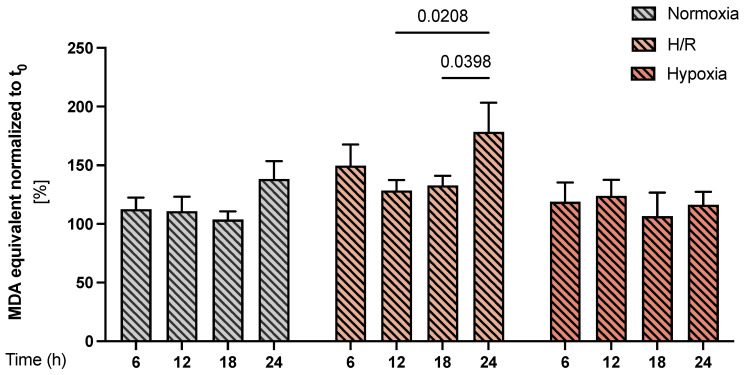
*Lipid peroxidation in the preeclampsia (PE) cell models.* The MDA equivalents were determined in the supernatant of the CTB cultures under normoxia (gray bars with diagonal lines), H/R (light orange bars with diagonal lines), and hypoxia (dark orange bars with diagonal lines) conditions using a thiobarbituric acid reactive substance (TBARS) assay during a time course of 6–24 h. For each condition, the results were normalized to the starting point (i.e., t = 0 h), by setting the time 0 h as 100%. Data are shown as the mean ± S.E.M (*n* = 9 different CTB isolations cultured under normoxia, H/R, and hypoxia). Statistical analyses were performed by paired 2-way ANOVA. *p* < 0.05 was considered statistically significant.

As a functional assessment of oxidative stress, we measured the level of malondialdehyde (MDA) equivalents, a major lipid peroxidation product, in the cell culture supernatants during the 6–24 h exposure to different oxygen conditions. We found significantly increased lipid peroxidation in H/R at 24 h compared with 12–18 h (*p* = 0.0208 and *p*= 0.0398) respectively in the same group; no significant changes over time were observed in the normoxia and hypoxia groups (Figure 6).

### 3.8. Protein Secretion of sFlT-1 and PlGF in PE Cell Models Reflect the Phenotype of PE

**Figure 7:**
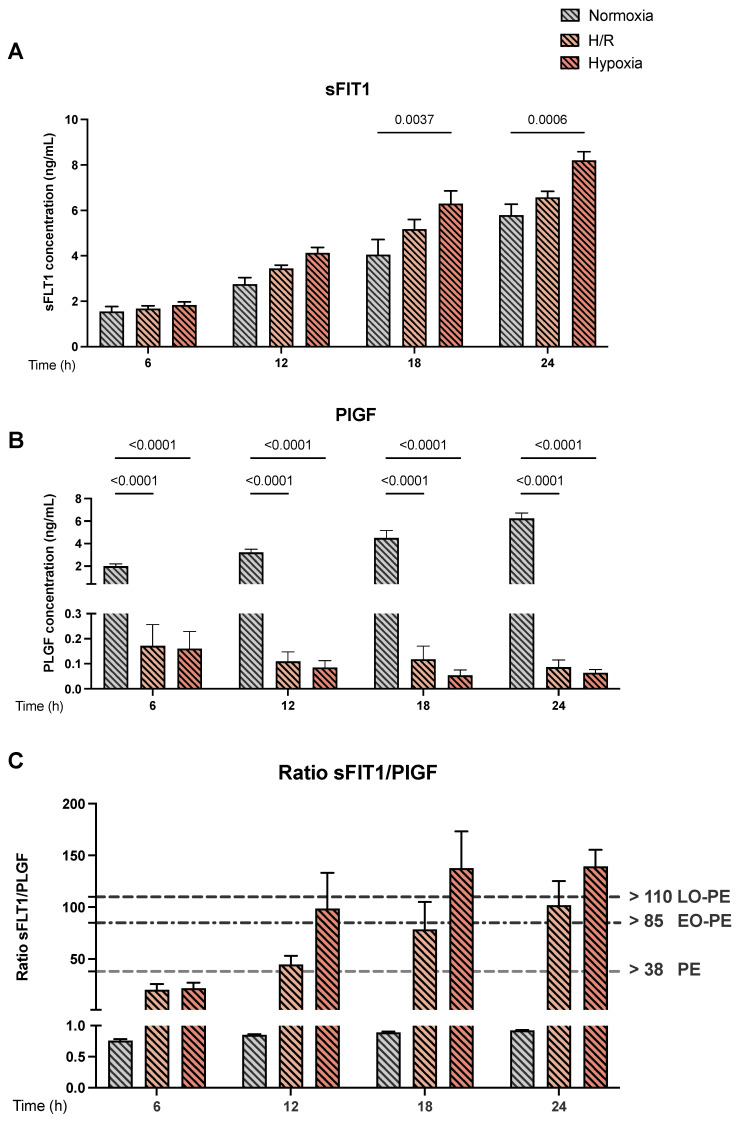
*The protein secretions of sFlT-1 and PlGF into the supernatants of the preeclampsia (PE) cell models*. The protein secretions of (**A**) sFlT-1 and (**B**) PlGF were measured using ELISA kits following the manufacturer´s instructions. The protein secretion was measured in the media of the CTB cultured under normoxia (gray bar with diagonal lines), H/R (light orange bar with diagonal lines), and hypoxia (dark orange bar with diagonal lines) conditions. (**C**) depicts the calculated sFlT-1/PlGF ratio. The gray dashed line (>38 PE) shows the cutoff value for PE as determined in human serum samples and used for diagnostic purposes in the clinical context. The black dash-dot line (>85) marks the cutoff value for early-onset PE (EO-PE), and the black dotted line (>110) depicts the cutoff value for late-onset PE (LO-PE). The results are presented as mean ± S.E.M. (*n* = 9 different CTB isolations cultured under normoxia, H/R, and hypoxia). Statistical analyses were performed by paired 2-way ANOVA. *p* < 0.05 was considered statistically significant.

Finally, we measured the release of sFlT-1 and PlGF by ELISA in the cell culture supernatants of the three cell models, i.e., under normoxic, H/R, and hypoxic conditions. We observed an increase in sFlT-1 release under hypoxia at 18 and 24 h (8.20 ± 0.38 and 6.30 ± 0.56 FC, (*p* = 0.0037 and *p* = 0.0006) respectively) compared to normoxia (Figure 7A). No differences were found for sFlT-1 secretion in H/R (Figure 7A). There was a significant reduction in PlGF secretion in H/R and hypoxia after 6 to 24 h of exposure to hypoxia compared with normoxia (*p* < 0.0001) (Figure 7B). Furthermore, for the H/R and hypoxia cell models, the calculated sFlT-1 to PlGF ratios exceeded the threshold of 38, a clinically used diagnostic parameter for PE [30,31], already after 12 h of exposure (Figure 7C). In fact, the calculated sFlT-1 to PlGF ratios in the H/R model exceeded the thresholds for early-onset PE (EO-PE; >85) after 18 h and the late-onset PE (LO-PE) thresholds (>110) after 24 h. Even more pronounced was the effect in the cell model under hypoxia alone where both EO-PE and LO-PE thresholds were already exceeded after 12 h of exposure.

## 4. Discussion

This novel study presents the thorough characterization of in vitro models for PE using primary cultures of trophoblast cells, exposed to hypoxia or H/R. 

It is believed that the alterations characterizing PE placentae are the result of chronic hypoxia [32]. However, comparison with morphological findings in other situations associated with low oxygen tensions such as IUGR and low birth weight suggests that hypoxia alone is insufficient to account for these changes [26]. The fetus and the placenta extract considerable oxygen during middle to late gestation, and the placental tissues will soon become locally hypoxic during periods of vasoconstriction [26,32]. When the maternal blood flow is re-established, there will be a rapid increase in tissue oxygenation. Such fluctuations in oxygen tension could provide the basis for an ischemia–reperfusion type insult [33,34,35]. Depending on the severity and frequency of these insults, the outcome might range from mild oxidative stress to severe tissue damage [26]. According to this information, we applied hypoxia and H/R in our study to determine if these two models resemble the PE phenotype and to assess the differential effects resulting from the different oxygen concentrations. 

The gene expression of the angiogenic factors FlT-1, ENG, and VEGF A were upregulated in the placentae of our PE cohort. These results agreed with other investigations performed under hypoxic conditions induced in isolated CTB [36,37]. The placental PlGF mRNA expression in our cohorts did not differ between the controls and women with PE. While a decrease in circulating PlGF levels in women with PE is manifested, the data for placental tissues are still unclear [38]. Indeed, a decrease due to persistent hypoxia and poor uteroplacental circulation [38,39], an elevation [40], and no changes [41] in PlGF expression in PE placental tissue have been reported. The discrepancies between these findings might be related to the heterogeneity of the women with PE with respect to their genetic background and the temporal onset of the disease. Interestingly, in the context of the measured angiogenic and antiangiogenic factors, the pure hypoxia model turned out to be more sensitive to the expected gene expression changes than H/R. These findings suggest that the trophoblast cells may compensate for the hypoxic damage during the six hours of reoxygenation.

It is well established that the RAS is also affected in PE [9,11,42,43]. Increased renin expression in human PE proposes activation of the uteroplacental RAS, which may lead or contribute to PE [11,42,43]. The results of the present study revealed no significant differences in mRNA abundances for AGTR1 either in the PE placenta samples or in the H/R or hypoxia models. However, it has been described that in PE, AGTR1 increases only in the decidua [44,45]. Considering that decidua was not included in the placental tissues used for this study, this might also explain the lack of differences found in our study. On the other hand, the placental AGTR4 mRNA levels were significantly reduced in the PE samples compared with the controls and in both hypoxia and H/R conditions compared with normoxia. Indeed, in agreement with our results, the placental expression of AGTR4 was found to be reduced in PE at term compared with the normotensive controls [46].

As outlined above, physiological hypoxia or low oxygen tension plays a critical role in early placental development. On the other hand, there is evidence that circulating plasma HIF1α levels are elevated in women with PE [47]. However, in human pregnancy a persistent hypoxic environment due to improper remodeling of the decidual spiral arteries will lead to an imbalanced angiogenic process, especially during the first two trimesters, contributing to the pathogenesis of PE [48,49]. In our experimental in vitro models, the mRNA expression of *HIF1α* remained unchanged under hypoxic conditions. These in vitro findings on *HIF1α* mRNA expression differ from our mRNA results in the PE placentae and other reports showing an increase in mRNA and protein of HIF1α in placental tissue from women with PE [9,49,50,51]. Though the reason for the lacking response in our experiments is unknown, it cannot be excluded that the effect of hypoxia on HIF1α is time-dependent and, hence, could have been missed in our experimental setup. Hence, the effect on HIF1α protein expression in term villi was previously observed after 7 h of hypoxia, while after 24 h of stimulation it was not detected anymore [52]. Thus, it would be worth analyzing HIF proteins under hypoxia and H/R conditions in tight time course experiments to elucidate the effect of hypoxia and H/R in this context in the future.

To further validate our cell models, we investigated the typical markers of oxidative stress, ER stress, and inflammation. The oxidative stress marker NRF2 was suggested to play a key role in PE [14,53,54,55] but with conflicting results regarding placental expression in women with PE [56,57]. In our study, the placental gene expression for *NRF2* was significantly reduced in PE and also in our cell models. NRF2 regulates the expression of multiple genes that encode detoxification enzymes and antioxidative proteins and protect the cells from oxidative stress [58]. A previous investigation demonstrated lower placental activation of NRF2 due to oxidative stress in women with PE [57]. This is in line with our findings and suggests that if NRF2 is less activated in trophoblasts, they fail to increase their antioxidant capacity thereby diminishing their cellular protection against oxidative stress.

Recent studies have also shown that the morphology of the ER is markedly altered in PE [59,60]. We detected differential expression levels of the ER stress markers GRP78 and GRP94. In the hypoxia PE cell model compared to normoxia, these findings were also supported by the results of the qPCR measurements in the PE placentae. However, H/R did not affect the expression of the ER stress markers, suggesting that this model reacts quickly and very sensitive to changes in oxygen, thus inducing fast recovery during the episodes of reoxygenation, restoring normal gene expression levels.

Previously, alterations in inflammatory markers such as IL-1β, caspase 1, and NLRP3 inflammasome have been described in PE both in the mother and the placenta [18,19,20]. Our studies revealed an increase in these three inflammasome markers in our PE cell models as well as in our PE placenta cohort, concurring with a previous placental study [19].

PP13 (also known as galectin-13) was also analyzed, as it has been suggested to be an early biomarker to assess the maternal risk for the subsequent development of pregnancy complications caused by impaired placentation [61]. Decreased placental expression of PP13 and low concentrations in the first trimester maternal sera were associated with an elevated risk of PE [62,63,64]. We found reduced gene expression of PP13 in hypoxic and H/R conditions compared to normoxia, underlining that the established cell models reflected to a high degree the in vivo situation. Similar to PP13, pregnancy-associated plasma protein (PAPP-A), a large highly glycosylated protein complex synthesized by trophoblasts [62], showed reduced gene expression in H/R. These results agree with the data demonstrating that decreased PAPP-A plasma or serum levels are associated with PE in the first trimester and throughout pregnancy [65,66,67].

Several studies on RGC32 revealed that this protein participates in cellular processes associated with cell differentiation, angiogenesis, migration, and invasion [68]. In our study, RGC32 was not significantly different in H/R and hypoxia. However, there are conflicting data as to whether PE and the associated hypoxia result in an increased or diminished expression of RGC32. Thus, Wang et al. showed by RT-PCR, western blotting, and immunohistochemistry that placental RGC32 expression was downregulated in PE compared to normotensive controls [68], confirming the reduced RGC32 levels in the PE samples presented in this study. 

hCG was evaluated in our study to assess the effect of hypoxia on trophoblast differentiation. Multiple studies have described a relation between high hCG levels and the risk of developing PE, negative effects on fetal development, and increased production of reactive oxygen species [69,70]. Additionally, high hCG levels were associated with increased production of sFlT-1 in women with PE [69]. We detected increased *hCG* mRNA expression in PE tissues, which agrees with other reports [69,70,71]. In contrast to the diminished *hCG* mRNA levels we found in both the H/R and the hypoxia model, hCG protein secretion was increased under hypoxia after 24 h. It has been previously described in the trophoblast cell lines JEG-3, BeWo, and JAr that hypoxia reduces the mRNA expression of *hCG* [72]. However, the detected changes in hCG secretion in the hypoxia cell model could be related to the de novo synthesis of hCG by trophoblast cells, which can adapt to a hypoxic environment [73], thus mimicking the phenotype of PE. 

Endothelial function in PE is affected by the oxidative stress-mediated excess of lipid peroxides [58,74], and increased lipid peroxide products were found in serum samples of preeclamptic women [75,76]. Our data showed continuous elevation and significantly increased MDA equivalents in H/R at 24 h compared to 12 and 18 h but not in the hypoxia model or normoxia control. Considering the levels of the oxidative damage of lipids, the H/R model seems to correspond better to the increased oxidative stress in PE.

Finally, we determined the clinically relevant parameters of sFLT-1 and PIGF in the supernatants of the hypoxic cell models. The protein secretion data showed the same trend as the gene expression results for FlT-1 in H/R and hypoxia and confirmed the significantly reduced PlGF levels in hypoxia in cell supernatants. In this context, Levine et al. showed that five weeks before the development of the clinical symptoms of PE, serum concentrations of sFLT-1 were elevated, and PLGF concentrations were decreased, resulting in an indicative increase in the sFLT-1/PLGF, ratio which can be used to predict the subsequent development of PE [30]. Recent evidence has also emerged that the ratio between sFlT-1 and PlGF in pregnant women’s serum could be used to predict early- and late-onset PE. In this context, it has been demonstrated that an sFlT-1/PlGF ratio > 85 for early-onset PE and >110 for late-onset PE represents a marker with a very high specificity of 99.5% and 95.5%, respectively [21]. To date, the sFlT-1/PlGF cutoff value ≤ 38 is widely accepted to rule out the assumptions for PE development [77]. Analogous to this clinical assessment, the sFlT-1/PlGF ratios determined in the cell supernatants of the hypoxia and H/R models were above 38 during the entire observation time. These data strongly suggest that the established cell models mimic to a marked extent the pathophysiological characteristics of PE. Therefore, we speculate that the development of novel therapeutic approaches can be envisioned.

In the current study we proposed a cell model for PE using primary CTB isolated from healthy term pregnancies. It is evident that the described in vitro models cannot fully reflect the complex mechanisms occurring during the pathogenesis of PE. Indeed, we focused in our studies on five main pathways known to be dysregulated in PE, but several additional markers could be investigated. Additionally, in PE, several cell types such as extravillous trophoblasts and vascular endothelial cells are supposed to be involved in the pathogenic processes, but we selectively investigated the contribution of the primary trophoblast cells. Moreover, for the disease phenotypes, the differentiation stage of the trophoblasts might play a major role (e.g., the degree of syncytium formation and the differentiation from CTB to EVT). For the sake of clarity and due to technical restrictions, these aspects were currently neglected and represent limitations of our studies.

In conclusion, our in vitro models with primary CTB, isolated from normal term pregnancy placentae and cultured under hypoxia or H/R, exhibited similar characteristics as placentae from women with PE. Thirteen of seventeen genes associated with angiogenesis, the renin–angiotensin system, oxidative stress, ER stress, and the inflammasome complex were susceptible to hypoxia and H/R mimicking the expression pattern of PE tissues. The sFlT-1/PlGF ratio obtained under hypoxia and H/R conditions confirms the results found in the serum from women with PE underlining the suitability of the models. In general, the two different oxygen conditions seem to complement each other and should be chosen depending on the pathways of interest to be investigated in PE. The characterized cell models can serve as a suitable tool for further investigation of PE, enabling the study of the underlying cellular mechanisms of this clinically severe pregnancy disease with still widely unclear pathogenesis.

## Figures and Tables

**Table 1:** Characteristics of primers.

Category	Gene	Accession Number	Primer Sequence (5′-3′)
Angiogenic and antiangiogenic markers	*FlT-1*	NM_001160030.1	F-AAGAGCCTGAACTGTATA R-TGATGACGATGATGATGA
	*ENG*	NM_001114753.2	F-CAAGACCAGGAAGTCCATA R-CGTGTGCGAGTAGATGTA
	*PIGF*	NM_001293643.1	F-CTCCTAAAGATCCGTTCTG R-CTTTCCGGCTTCATCTTC
	*VEGF A*	NM_001287044.1	F-TACATCTTCAAGCCATCC R-TTCTTTGGTCTGCATTCA
RAS receptors	*AGTR1*	NM_032049.3	F-TCTCAGCATTGATCGATACC R-TGACTTTGGCTACAAGCATT
	*AGTR4*	NM_005575.3	F-TATGCCTAAGAAGTCATCAG R-CCAAGTAAGTGCTCATCT
Hypoxia and oxidative stress markers	*HIF1α*	NM_001530.4	F-CGTTGTGAGTGGTATTATTC R-GGCTACTTGTATCTTCTGA
	*NRF2*	NM_005654.6	F-ATCGTGCTGTTCACGTCAGAC R-TGGCTCCTCACGTACTCCTC
Endoplasmic reticulum (ER) stress markers	*GRP78*	NM_005347.5	F-TGTGCAGCAGGACATCAAGT R-TCCCAAATAAGCCTCAGCGG
	*GRP94*	NM_003299.3	F-GCCAGTTTGGTGTCGGTTTC R-GGGTAATTGTCGTTCCCCGT
Placenta-specific proteins	*PP13*	NM_013268.3	F- GATATTGCCTTCCGTTTC R-GTAGTCTGTTGTCTCCTC
	*PAPP-A*	NM_002581.5	F-GCTGTCACATACATCCAT R-GCTGGGTTCATCAATACA
Cell cycle regulator	*RGC-32*	NM_014059.3	F-ATTCTCCAACAGACTCTAC R-CAAGATCAGCAATGAAGG
Syncytial marker	*β-hCG*	NM_033043.2	F-CGGGACATGGGCATCCAA R-GCGCACATCGCGGTAGTT
Inflammasome	*NLRP3*	NM_001079821	F-CACCTGTTGTGCAATCTGAAG R-GCAAGATCCTGACAACATGC
	*IL-1β*	NM_000576	F-CTGTCCTGCGTGTTGAAAGA R-TTGGGTAATTTTTGGGATCTAC
	*CASP1*	NM_033295	F-GCCTGTTCCTGTGATGTGGAG R-TGCCCACAGACATTCATACAGTTTC
Reference gene	*YWHAZ*	XM_024447266.1	F-CCGTTACTTGGCTGAGGTTG R-AGTTAAGGGCCAGACCCAGT

**Table 2:** Clinical characteristics of pregnant women and newborns.

Pregnancy Characteristics	Normal	Preeclampsia
Numbers	*n* = 25	*n* = 13
Maternal variables		
Age (years)	31.4 ± 4.9 (22–41)	31.7 ± 4.5 (24–42)
Length (cm)	163 ± 6.3 (155–176)	159.6 ± 7 (147–168)
Weight pre-pregnancy (kg)	61.7 ± 9.5 (45–86)	59.8 ± 10.6 (38–85)
Weight at delivery (kg)	73.3 ± 10 (58–102)	72.4 ± 10.8 (46–89)
BMI pre-pregnancy (kg/m^2^)	23 ± 2.4 (18.5–27.8)	23.4 ± 3.8 (17.6–33.6)
BMI at delivery (kg/m^2^)	27.5 ± 2.3 (23.2–32.9)	28.1 ± 3.8 (21.3–35-2)
Systolic blood pressure at delivery (mm Hg)	112.7 ± 10.9 (90–130)	155 ± 16.9 (138–185) *
Diastolic blood pressure at delivery (mm Hg)	70.9 ± 7 (60–88)	93.1 ± 10.4 (70–113) *
Basal glycemia (mg/dL)	77.6 ± 7.1 (66–92)	79 ± 11.2 (52–87)
Creatinine (mg/dL)	n.d.	115.8 ± 70.9 (26–236)
Proteinuria (mg/dL)	n.d.	2494 ± 1454 (150–19241)
Newborn variables		
Sex (female/male)	15/10	8/5
Gestational age (weeks)	39.1 ± 0.8 (38–40)	36.9 ± 2.1 (32–40) *
Route of delivery (C-section/Labor)	16/9	10/3
Birth weight (g)	3388 ± 367 (2765–4045)	2811 ± 866.2 (1030–3860) *
Height (cm)	50.2 ± 1.6 (48–53)	47.8 ± 4.7 (38–54) *
Ponderal index (g/cm^3^ x100)	2.7 ± 0.2 (2.3–3)	2.5 ± 0.3 (1.9–2.8) *

Weight and body mass index (BMI) were determined pregestationally and at the 3rd trimester; blood pressure was determined at delivery. n.d.: not determined. * *p* < 0.05 versus corresponding values in the PE group. Data are presented as the mean ± SD (range).

## Data Availability

The original contributions presented in the study are included in the article, further inquiries can be directed to the corresponding author.
